# A new method for anti‐negative interference of calcium dobesilate in serum creatinine enzymatic analysis

**DOI:** 10.1002/jcla.23928

**Published:** 2021-07-30

**Authors:** Hailan Shen, Kena Chen, Ju Cao

**Affiliations:** ^1^ Department of Laboratory Medicine The First Affiliated Hospital of Chongqing Medical University Chongqing China; ^2^ Key Laboratory of Diagnostic Medicine designated by the Ministry of Education Chongqing Medical University Chongqing China

**Keywords:** calcium dobesilate, creatinine, interference, negative, sarcosine oxidase enzymatic

## Abstract

**Background:**

Serum creatinine is a widely used biomarker for evaluating renal function. Sarcosine oxidase enzymatic (SOE) analysis is currently the most widely used method for the detection of creatinine. This method was negatively interfered with by calcium dobesilate, causing pseudo‐reduced results. The aim of this study was to explore a new method to alleviate the negative interference of this drug on creatinine detection.

**Method:**

We formulated eight drug concentrations and 12 creatinine concentrations from serum. The SOE method, the new method, and the Jaffe method were used for detection in five systems. Creatinine biases were analyzed under the conditions with or without the interference of calcium dobesilate, at consistent or inconsistent creatinine concentrations. Creatinine concentrations were also analyzed at three medical decision levels (MDLs).

**Results:**

Calcium dobesilate had negative interference in creatinine SOE analysis. With the increase in calcium dobesilate concentrations, the negative bias increases. The new BG method showed an anti‐negative interference effect. In the Roche system, the BG method reduced the negative bias from −71.11% to −16.7%. In the Abbott system, bias was reduced from −45.15% to −2.74%. In the Beckman system, the bias was reduced from −65.36% to −7.58%. In the Siemens system, the bias was reduced from −58.62% to −7.58%. In the Mindray system, the bias was reduced from −36.29% to −6.84%.

**Conclusion:**

The new method alleviated the negative interference of calcium dobesilate in creatinine SOE detection. The negative bias could be reduced from −60% or −70% to less than −20%.

## Graphical abstract

The serum creatinine quantitative method SOE was negatively interfered with by the commonly used drug calcium dobesilate, resulting in lower than the actual value. The new BG reagent laccase was added to prevent the generation of negative interference and make the detection more accurate. Schematic diagram of creatinine detection of the new BG reagent. SOD: sarcosine oxidase. 4‐AAP: 4‐aminophenazone. POD: peroxidase. HTIB: 2,4,6‐triiodo‐3‐hydroxybenzoic acid. Qic: quinoneimine chromogen. Calcium dobesilate is a strong reducing agent that consumes H_2_O_2_ during the reaction, resulting in decreased QIC of the final chromatographic substance. Laccase is a strong oxidant that can oxidize calcium dobesilate in advance before H_2_O_2_ is produced so that H_2_O_2_ will not be consumed and negative interference can be avoided.

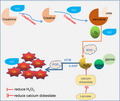

## 1 INTRODUCTION

Creatinine is a low‐molecular‐weight nitrogen‐containing compound, which is the end product of the metabolism of creatine and phosphocreatine.^1^, ^2^ Serum creatinine is the most widely used biomarker of renal function. It can be used to evaluate the glomerular filtration rate (GFR) and is considered as one of the main criteria for defining acute kidney injury (AKI).^3^ The accurate detection of serum creatinine is particularly important. According to the data from China National Clinical Laboratory Center, in 2015, creatinine was determined by enzymatic method and alkaline picric acid (APA), which accounted for 70.4% and 29.6% in Chinese laboratories, respectively. Enzymatic methods can be divided into the SOE method, creatinine amide hydrolase method, and imine hydrolase method according to different principles.^4^ The SOE method, also known as the enzyme method, is the most widely used creatinine detection method in China that was reported by Fossati et al in 1983. This method has the advantages of strong specificity, wide linear range, and strong anti‐interference; however, Trinder's reaction coupled with this method is susceptible to negative interference of reducing substances.

Calcium dobesilate is an oral microvascular protectant that improves abnormal hemorheology and microcirculation by reducing capillary permeability, blood viscosity, and platelet activity. It is widely used in the treatment of diabetic nephropathy, chronic renal insufficiency, coronary heart disease, and other diseases.^5^, ^6^, ^7^ It also has a therapeutic effect on gentamicin‐induced acute kidney injury.^8^ As a drug with strong reducing ability, calcium dobesilate has serious negative interference on enzymatic creatinine detection.^9^ Negative interference with calcium dobesilate has also been found in other Trinder's reaction–based tests for uric acid (UA), triglyceride (TG), total cholesterol (TC), glycated albumin (GA), and others, and its impact could not be underestimated.^10^ Therefore, clinical researchers are devoted to research and develop reagents that can have anti‐negative interference.

The aim of this study was to verify the anti‐interference effect of a newly developed enzymatic creatinine detection kit, try to solve the problem of false reduction on creatinine detection, and further the understanding of negative interference of drugs. As the reagent is cooperatively developed by the Zhongshan BGH Biochem. Co. Ltd., BG reagent and the BG method are used in this study to refer to the reagent.

## 2 MATERIALS AND METHODS

### 2.1 Instruments and reagents

Three different reagents were used to analyze creatinine concentration in five automatic biochemical analyzers in local laboratories: system‐matched SOE reagents, new enzymatic BG reagent, and APA reagent (the Jaffe principle). All five systems' matching reagents and calibrators are commercially available: Roche Creatinine Plus Reagent/Roche Cobas c701 Biochemical Analyzer (F. Hoffmann‐La Roche Ltd); Biosino Enzymatic Creatinine Reagent/Abbott ARCHITECT c16000 Biochemical Analyzer (Biosino, Bio‐Technology and Science Inc); Beckman Enzymatic Creatinine Reagent/Beckman AU5800 Biochemical Analyzer (Beckman Coulter, Inc); Siemens Enzymatic Creatinine Reagent/Siemens ADVIA XPT Biochemical Analyzer (Siemens); and Mindray Enzymatic Creatinine Reagent/Mindray BS2000M Biochemical Analyzer (Mindray Ltd). Five analyzers' parameter information and sampling patterns are shown in Tables S1‐S5. The new BG reagent and APA reagent were supplied by the company of Zhongshan BGH Biochem. Co., Ltd, with complete technical support. The new BG reagent was subjected to stability analysis before the experiment. Calcium dobesilate powder was obtained from Meryer Chemical Technology Co., Ltd. (Lot NO: 76827018). For instrument parameter settings, see Tables S1‐S5.

### 2.2 Serum collection and sample processing

Serum samples were collected from patients admitted to the First Affiliated Hospital of Chongqing Medical University between October 2019 and July 2020. Patients were required not to have taken any medication containing calcium dobesilate. The procedure was approved that this research was exempt from approval by the Chongqing Medical University Institutional Review Board and the Biomedical Ethics Committee. The samples were free of hemolysis, lipid blood, and jaundice. The creatinine mixture serum of each concentration gradient was collected. A total of 12 concentration gradients were collected (μmol/L): 40–59, 60–85, 86–100, 101–115, 116–133, 134–190, 191–250, 251–310, 311–370, 371–500, 501–600, and 601–720. The in vitro interference test was carried out according to EP7‐A2 by the American Institute for Clinical and Laboratory Standardization (CLSI).^11^ In order to ensure the reliability of the test data, the five tests were all carried out under the condition of internal quality control. All the prepared specimens were divided into five aliquots and frozen at −80°C before the test. All tests were repeated twice to get the average.

### 2.3 Preparation of calcium dobesilate in eight concentrations

Eight concentrations of calcium dobesilate were determined according to the pharmacokinetics: 0, 2, 4, 8, 16, 24, 32, 48, and 64 μg/ml.^12^ Concentration preparation method was as follows: The serum in each concentration of creatinine was first prepared to contain calcium dobesilate 65 μg/ml, and then, the concentration of 65 μg/ml was diluted with serum without calcium dobesilate to obtain other concentrations. In people, after a single oral 500 mg calcium dobesilate administration, after 3–6 h, blood drug concentration can reach the peak concentration of about 12.83–23.15 μg/ml and was maintained for 10 h.^13^ In human body, the trough drug concentration is 2.66–8.33 μg/ml, after which it slowly decreases and is undetectable after 24‐h administration. It was mainly excreted in urine and feces in the form of prototype, and about 10% of metabolites were excreted in urine within 24 h; the half‐life of the drug clearance is about 5 h. Within 5 min of intravenous administration of 500 mg, the plasma concentration reaches the peak value of 65 μg/ml and then rapidly decreases; the half‐time of plasma concentration is 1 h.^14^

### 2.4 Statistical analysis

All statistical analyses were performed using GraphPad Prism 8.0 software. It has been reported that calcium dobesilate does not interfere with the results of creatinine detection by analysis of alkaline picrate method, and the APA method can be used as an internal control for anti‐interference effect. The percentage deviations (*y*‐axis) were calculated based on the concentration of the drug‐free specimen without calcium dobesilate interference and were plotted vs. the calcium dobesilate concentrations (*x*‐axis) or vs. the creatinine concentrations (*x*‐axis).

## 3 RESULTS

### 3.1 Calcium dobesilate has obvious negative interference in creatinine enzymatic analysis in five auto‐biochemical analysis systems

**FIGURE 1:**
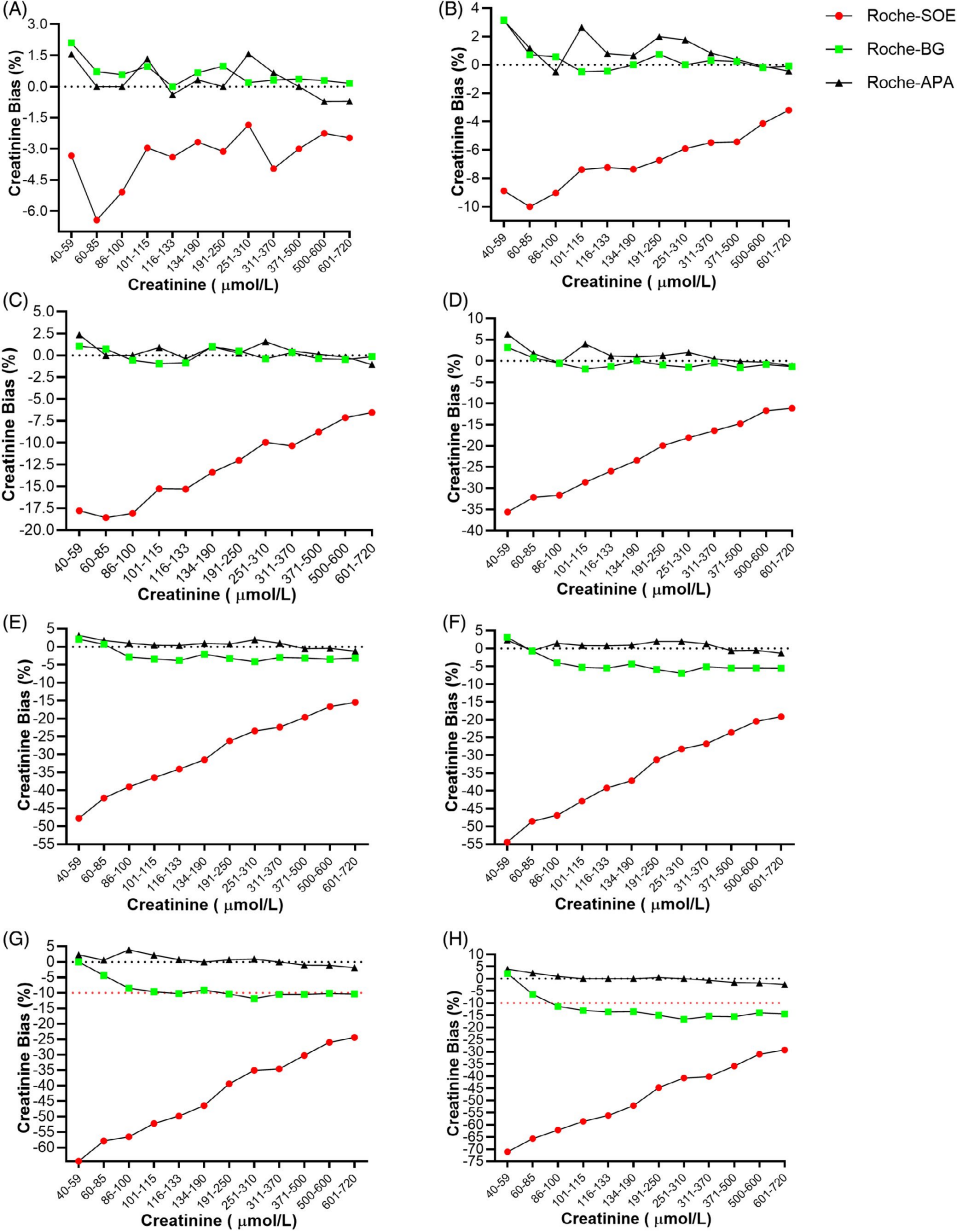
New BG reagent showed an anti‐negative interference effect on creatinine analysis in the Roche system. Three different reagents (system‐matched SOE reagent, new enzymatic BG reagent, and Alkaline picric acid [APA] reagent) were employed to detect creatinine concentration with the interference of calcium dobesilate at various concentrations ranging from 2 to 64 μg/ml. (A) 2 μg/ml calcium dobesilate. (B) 4 μg/ml. (C) 8 μg/ml. (D) 16 μg/ml. (E) 24 μg/ml. (F) 32 μg/ml. (G) 48 μg/ml. (H) 64 μg/ml

**FIGURE 2:**
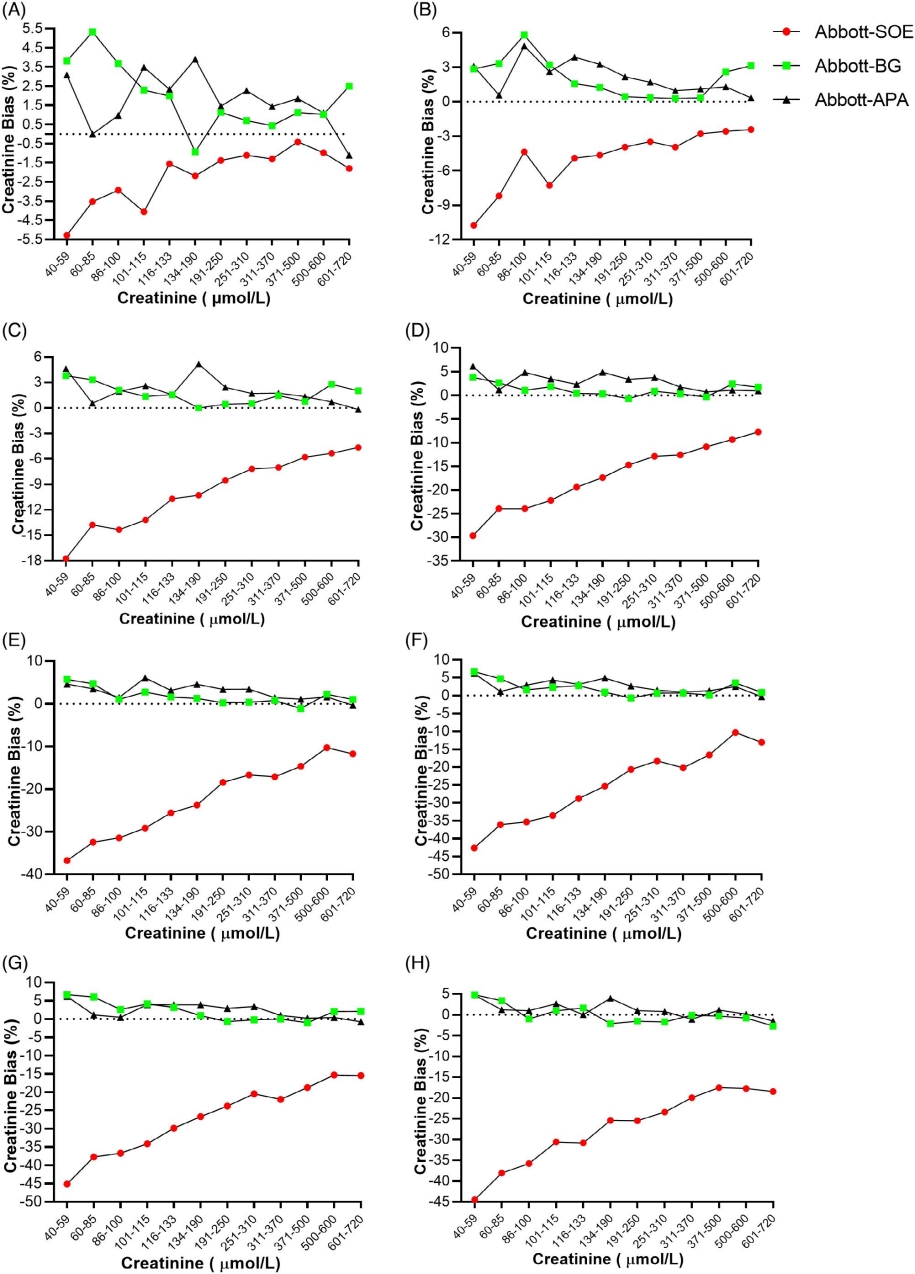
New BG reagent showed an anti‐negative interference effect on creatinine analysis in the Abbott system. Three different reagents (system‐matched SOE reagent, new enzymatic BG reagent, and alkaline picric acid [APA] reagent) were employed to detect creatinine concentration with the interference of calcium dobesilate at various concentrations ranging from 2 to 64 μg/ml. (A) 2 μg/ml calcium dobesilate. (B) 4 μg/ml. (C) 8 μg/ml. (D) 16 μg/ml. (E) 24 μg/ml. (F) 32 μg/ml. (G) 48 μg/ml. (H) 64 μg/ml

In every concentration, creatinine was negatively affected by calcium dobesilate in all five systems. In the Roche system, with the increase in concentrations of calcium dobesilate, the negative interference became more and more obvious (Figure 1). Interestingly, in a chart, with increased creatinine concentration, the interference effect decreased in the SOE method; that is, when creatinine concentration goes up, the negative interference by calcium dobesilate goes down. In the Roche system, negative interference bias ranged from −1.85% to −71.11%. The same situation was also observed in the Abbott system (Figure 2), where bias ranged from −0.41% to −45.15%; in the Beckman system (Figure S1), bias ranged from −1.54% to −64.78%; in the Siemens system (Figure S2), bias ranged from −0.67% to −58.62%; and in the Mindray system (Figure S3), bias ranged from −0.20% to −39%, revealing relatively lower negative interference compared with other systems.

### 3.2 New BG enzymatic method showed a superior advantage in anti‐negative interference of calcium dobesilate in five systems

**FIGURE 3:**
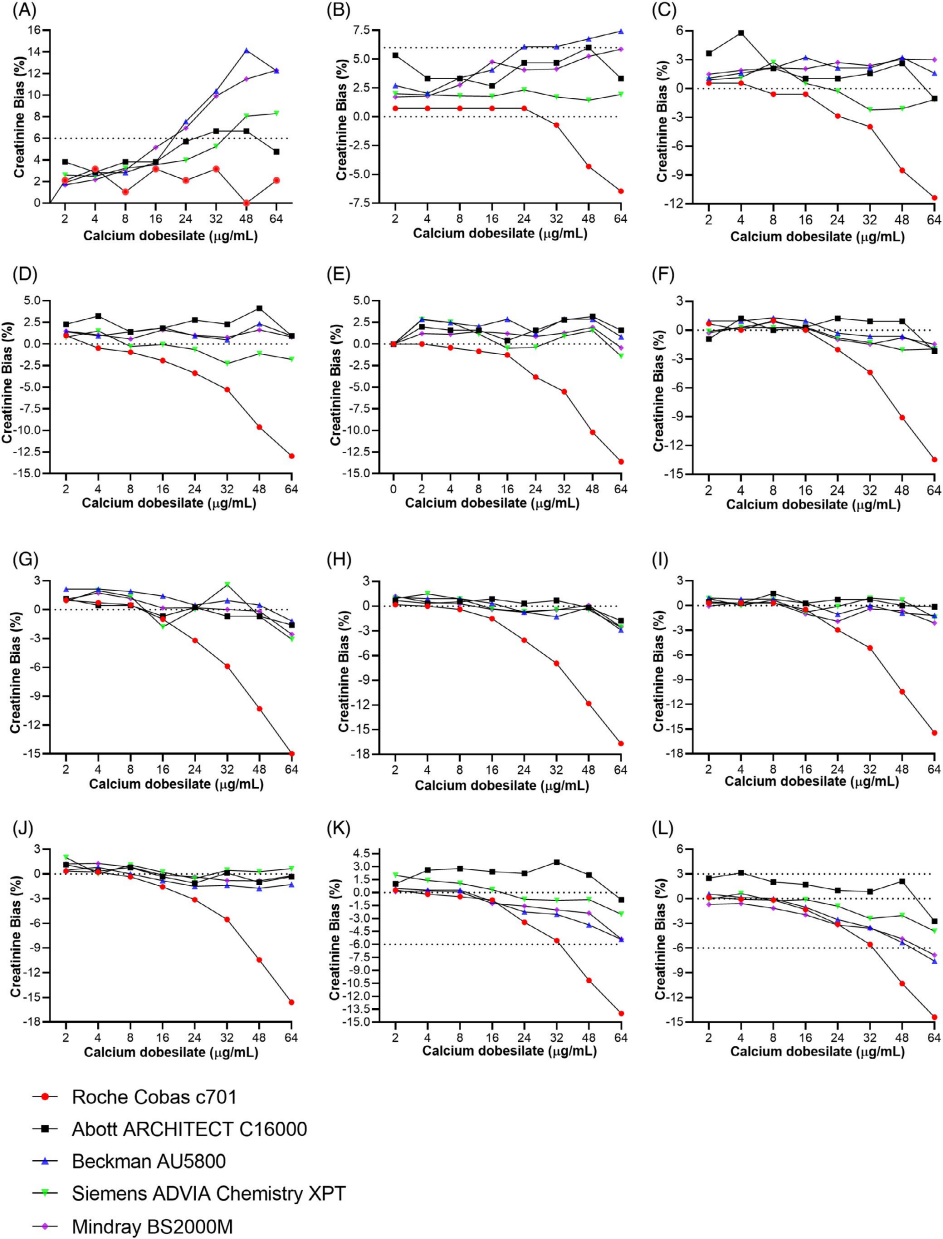
New BG method significantly reduced the negative interference of calcium dobesilate on creatinine detection. In five systems (Roche Cobas c701, Abbott ARCHITECT C16000, Beckman AU5800, Siemens ADVIA Chemistry XPT, and Mindray BS2000M) 12 creatinine concentrations were analyzed using BG new reagent. (A) 40–59 μmol/L creatinine concentration. (B) 60–85 μmol/L.(C) 86–100 μmol/L. (D) 101–115 μmol/L. (E) 116–133 μmol/L, (F) 134–190 μmol/L. (G) 191–250 μmol/L. (H) 251–310 μmol/L. (I) 311–370 μmol/L. (J) 371–500 μmol/L. (K) 501–600 μmol/L. (L) 601–720 μmol/L

**FIGURE 4:**
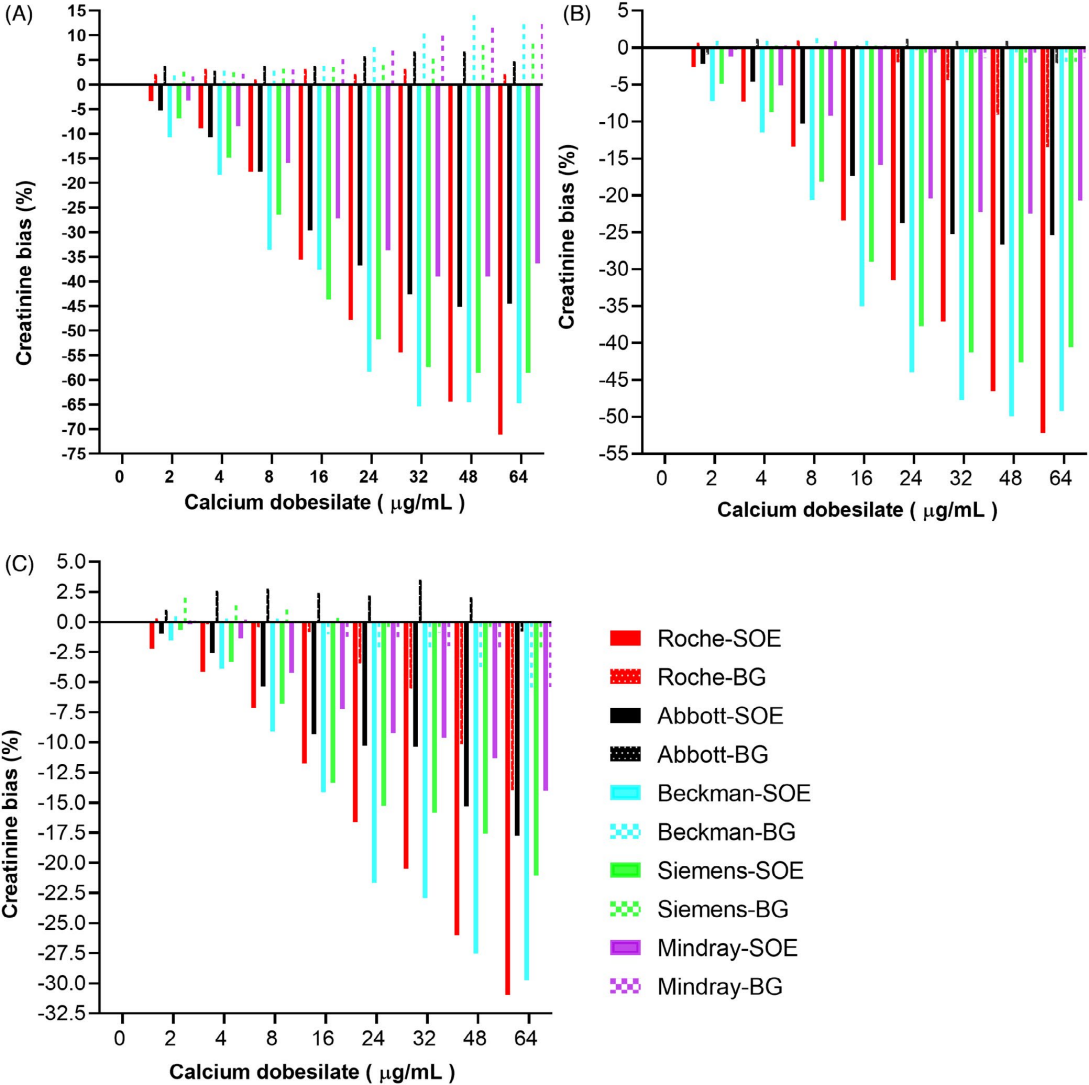
New method showed excellent anti‐negative interference of calcium dobesilate at creatinine medically determined levels. The creatinine values of the 3 medically determined levels (A) 40 μmol/L, (B) 141 μmol/L, and (C) 530 μmol/L were tested by the system‐matched SOE method and the new BG method in five systems under eight concentrations of calcium dobesilate

In five systems, we analyzed creatinine concentrations by using the BG method to compare with the original SOE method (matching system), where the new BG method showed obvious advantages (Figure 3). In the Roche system, the BG method reduced the negative bias from the maximum −71.11% (in 40–49 μmol/L creatinine concentration when calcium dobesilate is 64 μg/ml) to −16.7% (in 251–310 μmol/L creatinine concentration when calcium dobesilate is 64 μg/ml). In the Abbott system, the BG method reduced the negative bias from the maximum −45.15% (in 40–49 μmol/L creatinine concentration when calcium dobesilate is 48 μg/ml) to −2.74% (in 601–720 μmol/L creatinine concentration when calcium dobesilate is 64 μg/ml). In the Beckman system, the BG method reduced the negative bias from the maximum −65.36% (in 40–49 μmol/L creatinine concentration when calcium dobesilate is 32 μg/ml) to −7.58% (in 601–720 μmol/L creatinine concentration when calcium dobesilate is 64 μg/ml). In the Siemens system, the BG method reduced the negative bias from the maximum −58.62% (in 40–49 μmol/L creatinine concentration when calcium dobesilate is 64 μg/ml) to −7.58% (in 601–720 μmol/L creatinine concentration when calcium dobesilate is 64 μg/ml). In the Mindray system, the BG method reduced the negative bias from the maximum −36.29% (40–49 μmol/L creatinine concentration when calcium dobesilate is 64 μg/ml) to −6.84% (in 601–720 μmol/L creatinine concentration when calcium dobesilate is 64 μg/ml). Creatinine concentrations were also analyzed at three medical decision levels (MDL), and BG showed good anti‐interference effect (Figure 4).

## 4 DISCUSSION

A number of previous studies have reported on calcium dobesilate's interference with creatinine detection based on the Trinder reaction.^13^, ^15^, ^16^, ^17^ Because this reaction interferes with creatinine quantification, this is very detrimental to the evaluation of renal function, and the detection result is lower than the actual result due to negative interference.^15^ This can be interpreted as the patient's kidney function is improving or that the medication is responding, thus leading to a clinical misjudgment. Therefore, it is necessary to develop new reagents that can resist the negative interference of calcium dobesilate.

**FIGURE 5:**
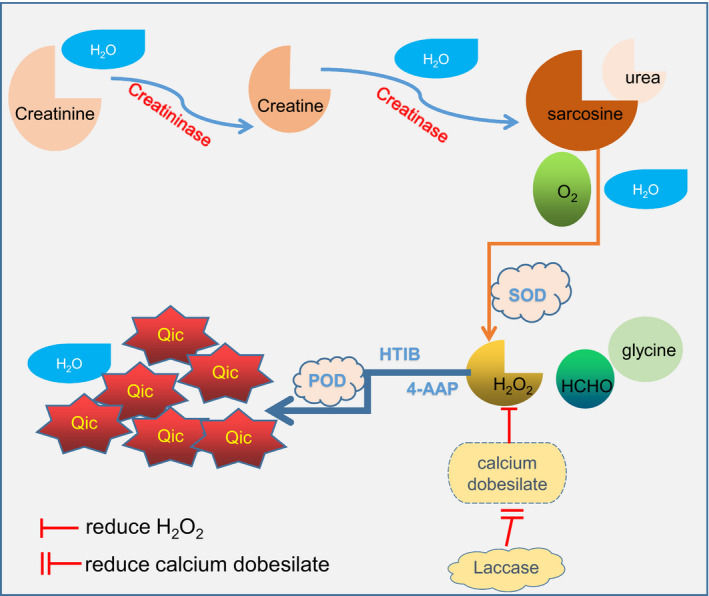
Schematic diagram of creatinine detection of the new BG reagent. SOD: sarcosine oxidase. 4‐AAP: 4‐aminophenazone. POD: peroxidase. HTIB: 2,4,6‐triiodo‐3‐hydroxybenzoic acid. Qic: quinoneimine chromogen. Calcium dobesilate is a strong reducing agent that consumes H_2_O_2_ during the reaction, resulting in decreased QIC of the final chromatographic substance. Laccase is a strong oxidant that can oxidize calcium dobesilate in advance before H_2_O_2_ is produced so that H_2_O_2_ will not be consumed and negative interference can be avoided

When creatinine concentration was detected by the SOE method, hydrogen peroxide (H_2_O_2_) promoted 4‐amino‐aminopyrine (4‐AAP) reaction to produce quinoneimine chromogenic agent. The color intensity of the resulting quinoneimine chromogen is proportional to the creatinine concentration. However, as H_2_O_2_ is a strong oxidant in this process, it can be easily interfered by strong reducing substances. Calcium dobesilate is a potent reducing drug, which reduces H_2_O_2_ and causes a reduction in color‐developing substances, which, in turn, leads to the pseudo results and the formation of negative interference. This is an essential factor in the results of all Trinder's chemical reaction patterns. In collaboration with the reagent company, we developed the new enzymatic method: BG, laccase was added to reagent 1. Laccase is a strong oxidizing substance that can oxidize calcium dobesilate before H_2_O_2_ is produced to prevent it from degrading H_2_O_2_ in reaction and achieve an anti‐negative interference effect. A schematic diagram of creatinine detection of the new BG method is shown in Figure 5.

The new reagent had different anti‐negative interference effects, but it was not perfect in five automatic biochemical analyzers. When calcium dobesilate exceeded 48 μg/ml in the Roche system, there was still negative bias, and the negative interference became more obvious with the increase in creatinine concentration (Figure 3). Consequently, this new method was not suitable for Roche cobas c701 when calcium dobesilate was above 32 μg/ml. In response to this phenomenon, we consulted some application engineers who suggested that this may be due to parameter settings, when performing BG new reagent testing on Roche Cobas 701, the first absorbance reading dot maybe should not be set to the 19th point; the Roche kit is the first calculated absorbance value read at the 22nd point to eliminate some background interference (Table S1). As can be seen from Figure 3, the anti‐negative interference effect was effective when the calcium dobesilate was in low concentration. We wondered whether there were not enough anti‐interference substances. Accordingly, we further explored the concentration of laccase to test whether increasing the amount of it would work in the Roche system. It is necessary to continue exploring the ingredient of reagents 1 and 2 to get a better formula and further explore the ratio of various components in anti‐interference reagents. Even though we tried this new method in five systems, many systems have not been tested yet; thus, it remains unknown whether this method might be suitable or not in other analytic systems.

Drug laboratory test interactions (DLTIs) are one of the major sources of laboratory errors, and it is necessary to strengthen the publicity and education of drug interference and enhance the attention of doctors and patients to drug interference on blood tests.^16^ In addition to the calcium dobesilate highlighted in this article, there are also ethamsylate, phenolic sulfoethylamine, acetaminophen, ascorbic acid, catecholamine, aspirin, dopamine, analgin, and rifampicin detection inferenced by color development.^17^, ^18^, ^19^, ^20^ Trinder's reaction involves not only creatinine but also uric acid (UA), triglyceride (TG), cholesterol (TC), high‐density lipoprotein cholesterol (HDL‐C), low‐density lipoprotein cholesterol (LDL‐C), etc.^13^, ^21^ many clinical drugs' interference tests are included in the performance evaluation of a detected item, but cannot cover all kinds of drugs. When judging test results, clinicians need to analyze the patient's condition from many aspects, and it is necessary to integrate multiple items of the same clinical significance for comparison so as to reach a more scientific conclusion. At the same time, due to the differences between the detection systems, it is recommended not to frequently change the detection methods and detection systems when judging the disease or efficacy.

## CONFLICT OF INTEREST

All authors declare that they have no potential conflicts of interest. There is no commercial conflict of interest in this article. The authors declare that the research was conducted in the absence of any commercial or financial relationships that could be construed as a potential conflict of interest.

## AUTHOR CONTRIBUTIONS

Hailan Shen contributed to this research design, data acquisition and interpretation, graph and table making, and writing and submitting this article. Kena Chen contributed to this research design, specimen collection, specimen testing, and data analysis. Dr Ju Cao contributed to the conception and design of the research and manuscript review.

## Supporting information

Fig S1

Fig S2

Fig S3

Table S1‐S5

Supplementary Material

## Data Availability

All data are included in this article.
