# Supplementary figures and images for: A new method for anti‐negative interference of calcium dobesilate in serum creatinine enzymatic analysis
Source: J Clin Lab Anal. 2021 Jul 30;35(9):e23928. doi: 10.1002/jcla.23928 (PMC8418471; doi:10.1002/jcla.23928)

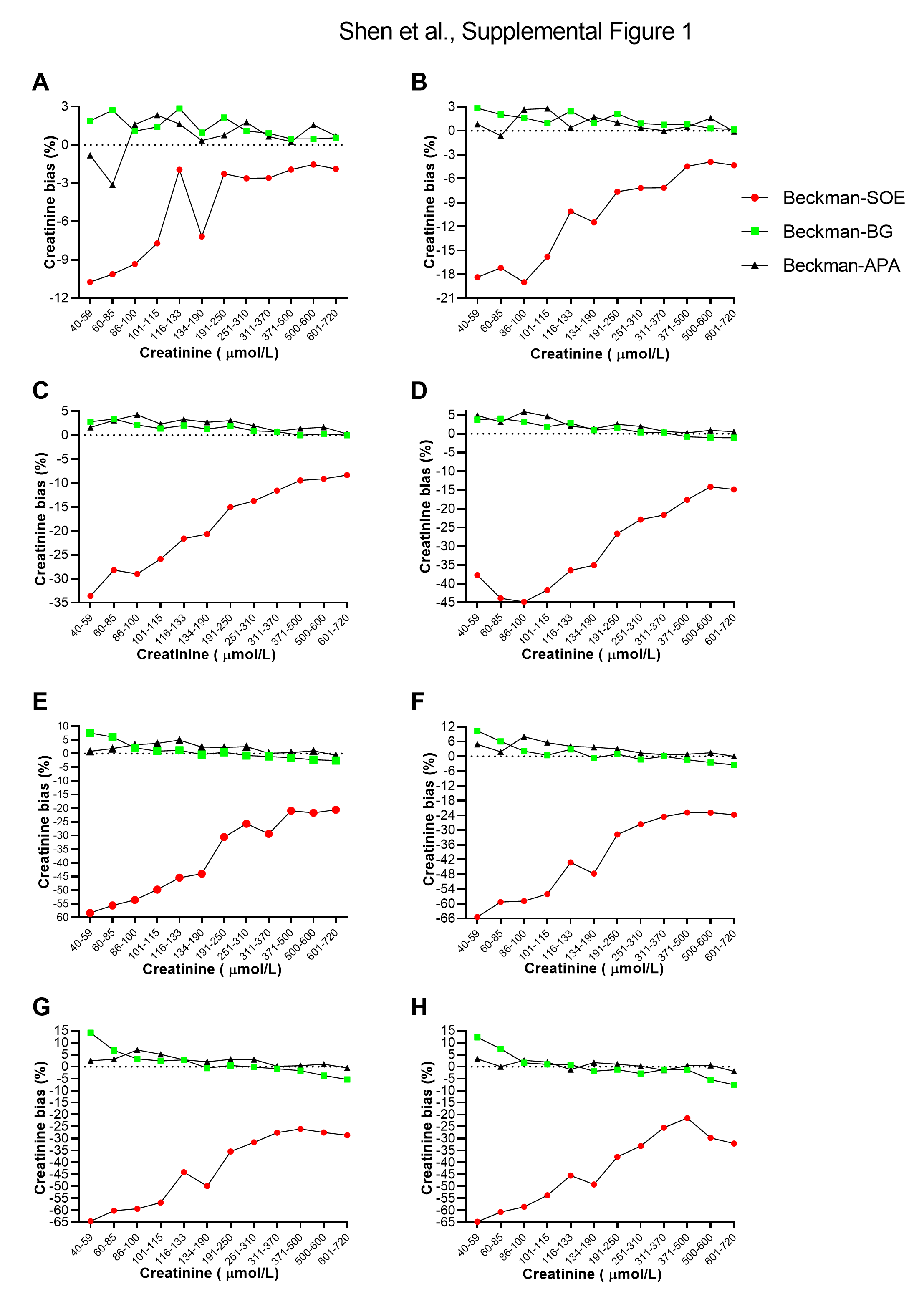

Supplement: Supplementary file 1 — Fig S1 [file JCLA-35-e23928-s004.tif]

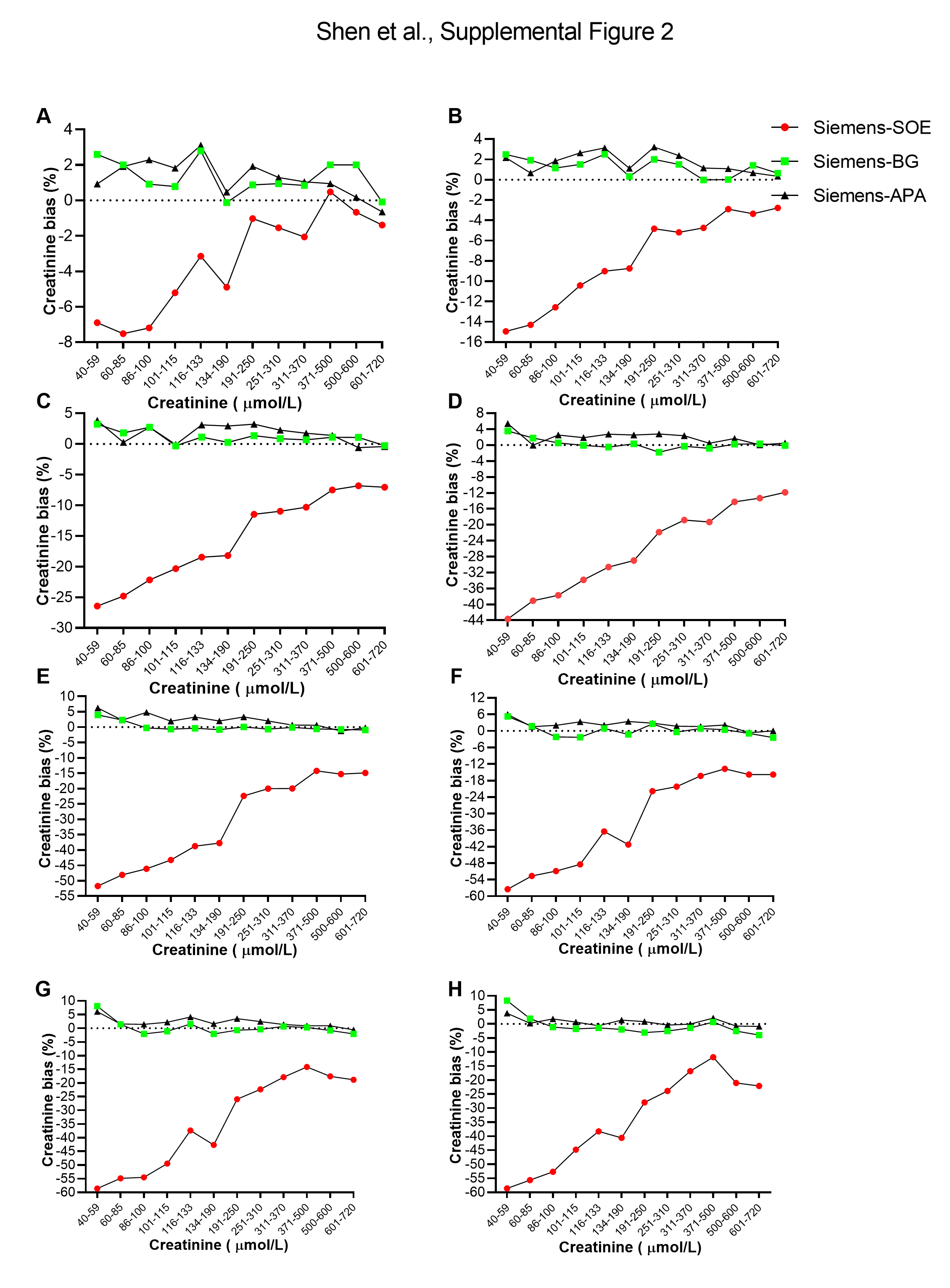

Supplement: Supplementary file 2 — Fig S2 [file JCLA-35-e23928-s002.tif]

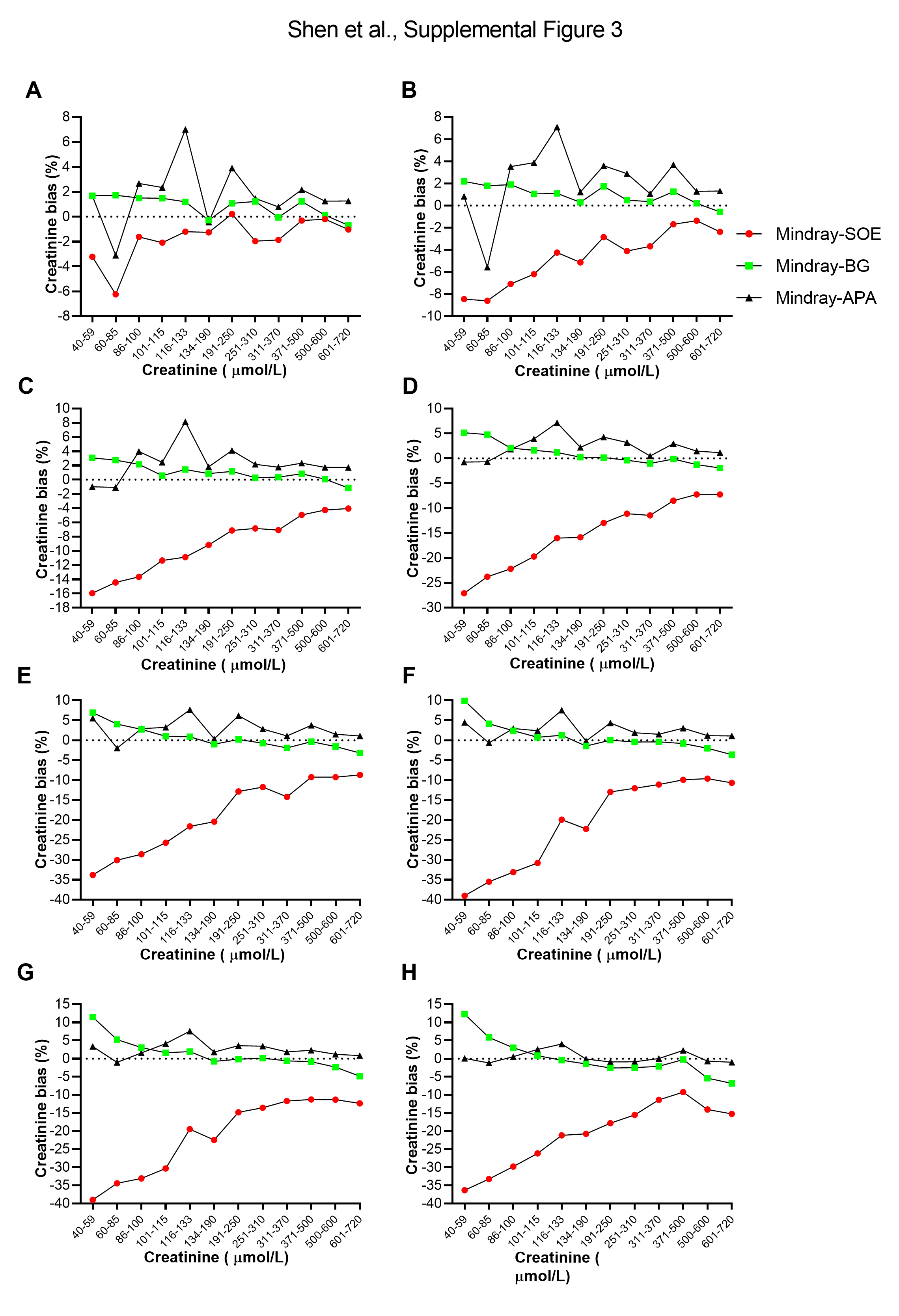

Supplement: Supplementary file 3 — Fig S3 [file JCLA-35-e23928-s001.tif]
