# Supplementary material for: A new method for anti‐negative interference of calcium dobesilate in serum creatinine enzymatic analysis
Source: J Clin Lab Anal. 2021 Jul 30;35(9):e23928. doi: 10.1002/jcla.23928 (PMC8418471; doi:10.1002/jcla.23928)
Supplement: Supplementary file 4 — Table S1‐S5 [file JCLA-35-e23928-s005.docx]

**Supplemental Table 1 Roche cobas c701 parameters**

|  | SOE | BG | APA |
| --- | --- | --- | --- |
| Reaction model | 2-point endpoint | 2-point endpoint | 2- point rate |
| Reaction time | 10min | 10min | 10min |
| Assay points | 22, 38 | 19, 38 | 21, 25 |
| Sub wavelength | 700 | 660 | 660 |
| Main wavelength | 546 | 546 | 505 |
| Sample (μL) | 2.0 | 8.0 | 7 |
| R1 (μL) | 77 | 168 | 168 |
| R2 (μL) | 38 | 56 | 42 |

**Supplemental Table 2 Abbott ARCHITECT C16000 parameters**

|  | SOE | BG | APA |
| --- | --- | --- | --- |
| Reaction model | End up | End up | Rate increase |
| Main/sub wavelength | 548/660 | 548/660 | 524/660 |
| Main Assay point | 31-33 | 31-33 | 18-20 |
| Final Assay point | 33 | 33 | 33 |
| Sample blank type | Self blank | Self blank | no |
| Blank read time | 14-16 | 14-16 | - |
| Reagent R1/R2(μL) | 162/81 | 168/56 | 168/42 |
| Sample(μL) | 4.3 | 8.0 | 7.0 |

**Supplemental Table 3 Beckman AU5800 parameters**

|  | SOE | BG | APA |
| --- | --- | --- | --- |
| Method | Endpoint | Endpoint | Rate method |
| Main/sub wavelength | 600/700 | 540/660 | 520/660 |
| Analyze dots 1 | Initial: 0, final: 27 | Initial: 10, final: 27 | Initial: 11, final: 13 |
| Analyze dots2 | Initial: 0, final: 10 | - | - |
| R1/R2(μL) | 90/30 | 168/56 | 168/42 |
| Sample(μL) | 4.2 | 8.0 | 7.0 |
| （Sample）dilution(μL) | 10 | 0 | 0 |

**Supplemental Table 4 Siemens ADVIA Chemistry XPT parameters**

|  | SOE | BG | APA |
| --- | --- | --- | --- |
| Method | Endpoint | Endpoint | Rate method |
| Reaction time | 10min | 10min | 10min |
| Main/sub wavelength | 596/694 | 545/658 | 505/658 |
| Reaction analyze dot (main analyze) | Initial:39, Final:41 | Initial:40, Final:41 | Initial:23,  Final:25 |
| Reaction Assay points (Secondary analyze) | Initial:20, final:21 | Initial:19, final:21 | - |
| Sample(μL) | 13.0 | 3.0 | 3.0 |
| Dilution method | standard | - | - |
| Dilution: sample volume(μL) | 30 | - | - |
| Dilution buffer(μL) | 120 | - | - |

**Supplemental Table 5 Mindray BS2000M parameters**

|  | SOE | BG | APA |
| --- | --- | --- | --- |
| Method | Endpoint | Endpoint | Fixed time |
| Main/sub wavelength | 546/700 | 546/660 | 505/660 |
| Reagent R1/R2(μL) | 150/50 | 168/56 | 156/42 |
| Sample(μL) | 5.0 | 8.0 | 7.0 |
| Blank time | 14-16 | 14-16 | — |
| Reaction time | 31-33 | 31-33 | 17-19 |

Table S1-5 Note: SOE: sarcosine oxidase enzymatic method, BG: enzymatic new method, APA: Alkaline picric acid method, R1: Reagent 1, R2: Reagent 2.
