# Supplementary material for: A new method for anti‐negative interference of calcium dobesilate in serum creatinine enzymatic analysis
Source: J Clin Lab Anal. 2021 Jul 30;35(9):e23928. doi: 10.1002/jcla.23928 (PMC8418471; doi:10.1002/jcla.23928)
Supplement: Supplementary file 5 — Supplementary Material [file JCLA-35-e23928-s003.docx]

**SUPPLEMENTARY MATERIALS**

**Figure S1.** **The new BG reagent showed an anti-negative interference effect on creatinine analysis in the Beckman system.** Three different reagents (system-matched SOE reagent, new enzymatic BG reagent and Alkaline picric acid (APA) reagent) were employed to detect creatinine concentration with the interference of calcium dobesilate at various concentrations ranging from 2-64 μg/mL. (A) 2 μg/mL calcium dobesilate. (B) 4 μg/mL. (C) 8 μg/mL. (D) 16 μg/mL. (E) 24 μg/mL. (F) 32 μg/mL. (G) 48 μg/mL. (H) 64 μg/mL.

**Figure S2. The new BG reagent showed an anti-negative interference effect on creatinine analysis in the Siemens system.** Three different reagents (system-matched SOE reagent, new enzymatic BG reagent and Alkaline picric acid (APA) reagent) were employed to detect creatinine concentration with the interference of calcium dobesilate at various concentrations ranging from 2-64μg/mL. (A) 2 μg/mL calcium dobesilate. (B) 4 μg/mL. (C) 8 μg/mL. (D) 16 μg/mL. (E) 24 μg/mL. (F) 32 μg/mL. (G) 48 μg/mL. (H) 64 μg/mL.

**Figure S3. The new BG reagent showed an anti-negative interference effect on creatinine analysis in the Mindray system.** Three different reagents (system-matched SOE reagent, new enzymatic BG reagent and Alkaline picric acid (APA) reagent) were employed to detect creatinine concentration with the interference of calcium dobesilate at various concentrations ranging from 2-64 μg/mL. (A) 2 μg/mL calcium dobesilate. (B) 4 μg/mL. (C) 8 μg/mL. (D) 16 μg/mL. (E) 24 μg/mL. (F) 32 μg/mL. (G) 48 μg/mL. (H) 64 μg/mL.
